# A *mir-231*-Regulated Protection Mechanism against the Toxicity of Graphene Oxide in Nematode *Caenorhabditis elegans*

**DOI:** 10.1038/srep32214

**Published:** 2016-08-25

**Authors:** Ruilong Yang, Mingxia Ren, Qi Rui, Dayong Wang

**Affiliations:** 1 College of Life Sciences, Nanjing Agricultural University, Nanjing 210095, China; 2 Key Laboratory of Environmental Medicine Engineering in Ministry of Education, Medical School, Southeast University, Nanjing 210009, China

## Abstract

Recently, several dysregulated microRNAs (miRNAs) have been identified in organisms exposed to graphene oxide (GO). However, their biological functions and mechanisms of the action are still largely unknown. Here, we investigated the molecular mechanism of *mir-231* in the regulation of GO toxicity using *in vivo* assay system of *Caenorhabditis elegans*. We found that GO exposure inhibited the expression of *mir-231::GFP* in multiple tissues, in particular in the intestine. *mir-231* acted in intestine to regulate the GO toxicity, and overexpression of *mir-231* in intestine caused a susceptible property of nematodes to GO toxicity. *smk-1* encoding a homologue to mammalian SMEK functioned as a targeted gene for *mir-231*, and was also involved in the intestinal regulation of GO toxicity. Mutation of *smk-1* gene induced a susceptible property to GO toxicity, whereas the intestinal overexpression of *smk-1* resulted in a resistant property to GO toxicity. Moreover, mutation of *smk-1* gene suppressed the resistant property of *mir-231* mutant to GO toxicity. In nematodes, SMK-1 further acted upstream of the transcriptional factor DAF-16/FOXO in insulin signaling pathway to regulate GO toxicity. Therefore, *mir-231* may encode a GO-responsive protection mechanism against the GO toxicity by suppressing the function of the SMK-1 - DAF-16 signaling cascade in nematodes.

Graphene oxide (GO) is a carbon-based, two-dimensional engineered nanomaterial (ENM) with a high coefficient of thermal conduction and large surface area that is chemically stable, amphipathic and easy to be functionalized^1^. These properties make GO very attractive for several commercial and medical applications, including drug delivery and bioimaging^2^,^3^. However, toxicological studies in mammals have shown that GO exposure could result in the induction of oxidative stress and organ system dysfunctions such as pulmonary and reproductive toxicity^4^,^5^. The classic model animal of nematode *Caenorhabditis elegans* is an important non-mammalian alternative for toxicological study^6^,^7^. Previous studies using nematodes have demonstrated that GO exposure could lead to toxic effects on the functions of both primary (such as intestine) and secondary (such as neuron and reproductive organs) targeted organs^8^,^9^,^10^,^11^,^12^,^13^. Furthermore, the activation of oxidative stress, enhanced intestinal permeability, disrupted innate immune response, and prolonged defecation cycle length were found to contribute to the formation of GO toxicity in nematodes^9^,^10^,^11^.

So far, the underlying molecular mechanisms of GO toxicity have for the most part remained elusive. Recent studies have implicated specific signaling pathways, including the Toll-like receptor 4 (TLR4) and the c-Jun N-terminal kinase (JNK) signaling pathways, in the control of GO toxicity in macrophages or nematodes^14^,^15^. Moreover, some dysregulated mRNAs or microRNAs (miRNAs) have been identified in GO exposed human HepG2 and GLC-82 cells as well as in nematodes^16^,^17^,^18^. Short noncoding miRNAs exist in many organisms and usually act post-transcriptionally to inhibit the expression of targeted genes^19^. Therefore, the characterization of candidate miRNAs and their roles in regulating GO toxicity will further improve our understanding of the underlying molecular mechanisms of GO toxicity.

Our previous studies have demonstrated that GO exposure resulted in the dysregulation of 1965 mRNAs and 31 miRNAs in nematodes^15^,^18^. One of these dysregulated miRNAs, *mir-231*, was down-regulated in response to GO exposure, and mutation of *mir-231* induced a resistant property to GO toxicity in nematodes^18^. In nematodes, *mir-231* is expressed from embryonic through adult stages, and is expressed in the intestine, pharynx, hypodermis, and neurons in adults^20^,^21^. So far, the biological functions of *mir-231* are still largely unknown. In the present study, we employed the *in vivo* assay system of *C. elegans* to investigate the molecular mechanisms of *mir-231* in regulating GO toxicity. The *C. elegans* protein SMK-1 is orthologous to mammalian SMEK (suppressor of MEK null) and essential for the function of DAF-16-mediated longevity^22^. *daf-16* gene encodes the transcriptional factor DAF-16/FOXO in the insulin signaling pathway^23^. Our results suggest that *mir-231* may regulate GO toxicity by suppressing the function of SMK-1-DAF-16 signaling cascade in nematodes. Our study underlines the importance of *mir-231* in encoding a protection mechanism against GO toxicity.

## Results

### Physicochemical properties of GO

**Figure 1:**
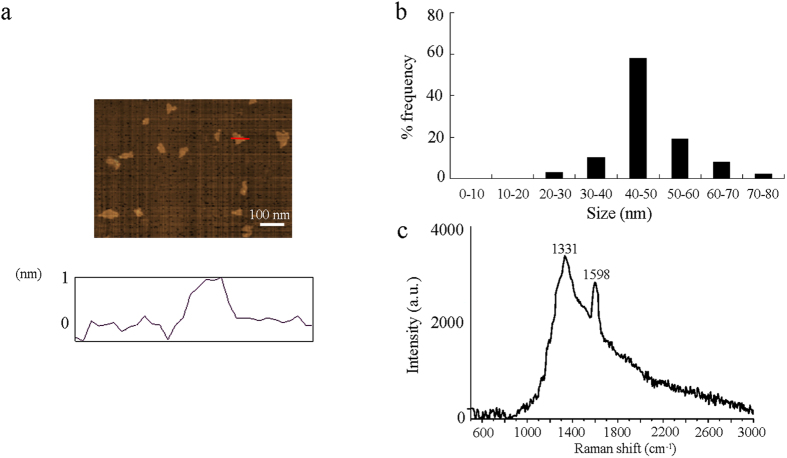
Physiochemical properties of GO. (**a**) AFM analysis of GO. (**b**) Size distribution of GO. (**c**) Raman spectrum of GO.

The thickness of GO was approximately 1.0 nm in topographic height, corresponding to the property of approximately one layer (Fig. 1a). Sizes of most of the GO in K-medium after sonication were in the range of 40–50 nm (Fig. 1b). Raman spectroscopy assay showed the existence of D-band signal of GO, suggesting the introduction of disorder into graphite layer (Fig. 1c). In Raman spectroscopy, GO showed a G band at 1598 cm^−1^ and a D band at 1331 cm^−1^, respectively (Fig. 1c). Zeta potential of GO (100 mg/L) in K-medium was −21.5 ± 2.6 mV.

### Effect of GO exposure on spatial expression of *mir-231* in nematodes

**Figure 2:**
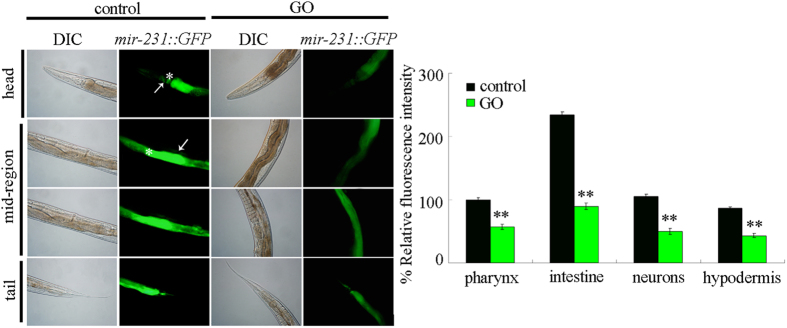
Effects of GO exposure on *mir-231::GFP* expression in nematodes. Asterisks indicate the pharynx and intestine in the head and mid-region, respectively. Arrowheads indicate the neurons and hypodermis in the head and mid-region, respectively. GO exposure concentration was 100 mg/L. Prolonged exposure was performed from L1-larvae to young adults. Bars represent means ± SD. **P < 0.01 *vs* control.

Using transgenic strain of *maIs218*, we investigated the effect of GO exposure on spatial expression of *mir-231::GFP* in nematodes. *mir-231::GFP* is expressed in pharynx, intestine, neurons, and hypodermis (Fig. 2). Especially, *mir-231::GFP* is predominantly expressed in intestine (Fig. 2). GO at the concentration of 100 mg/L caused the reduction in lifespan, decrease in locomotion behavior, and significant induction of reactive oxygen species (ROS) production in nematodes^9^. After prolonged exposure, we observed that GO (100 mg/L) significantly decreased the fluorescence intensity of *mir-231::GFP* in pharynx, intestine, neurons, and hypodermis compared with control (Fig. 2). Moreover, the more sharp reduction in fluorescence intensity of *mir-231::GFP* was observed in intestine of GO (100 mg/L) exposed nematodes (Fig. 2).

### Tissue-specific activity of *mir-231* in regulating GO toxicity in nematodes

**Figure 3:**
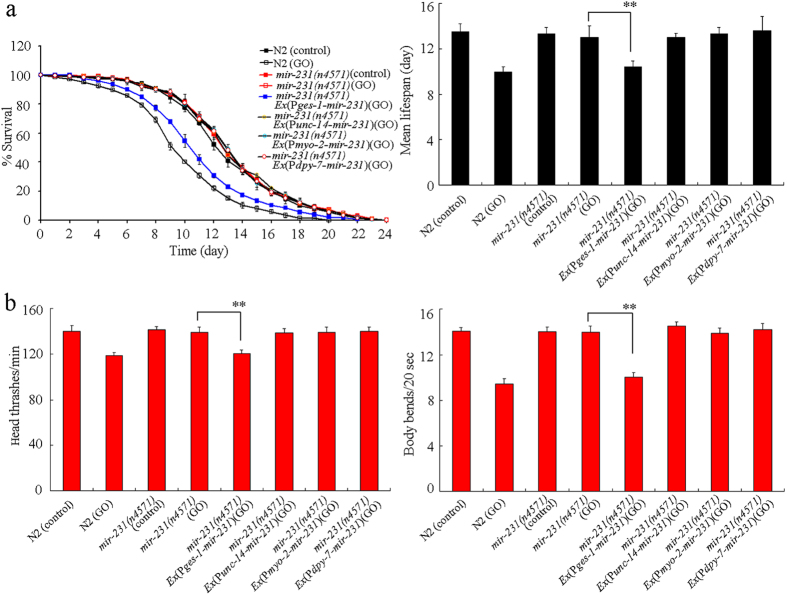
Tissue-specific activity of *mir-231* in regulating GO toxicity in nematodes. (**a**) Tissue-specific activity of *mir-231* in regulating GO toxicity on lifespan in nematodes. (**b**) Tissue-specific activity of *mir-231* in regulating GO toxicity on locomotion behavior in nematodes. GO exposure concentration was 100 mg/L. Prolonged exposure was performed from L1-larvae to young adults. Bars represent means ± SD. **P < 0.01.

Using tissue-specific promoters, we next investigated the tissue-specific activity of *mir-231* in regulating GO toxicity in nematodes. *mir-231*(*n4571*) mutant had a normal lifespan and locomotion behavior (Fig. 3). Loss-of-function mutation of *mir-231* induced a resistant property to GO toxicity on lifespan and locomotion behavior in nematodes (Fig. 3). Rescue assay by expression of *mir-231* in the neurons, pharynx, or hypodermis did not significantly affect the resistant property to GO toxicity on lifespan and locomotion behavior in *mir-231*(*n4571*) mutant nematodes (Fig. 3). In contrast, expression of *mir-231* in the intestine significantly suppressed the resistant property to GO toxicity on lifespan and locomotion behavior in *mir-231*(*n4571*) mutant nematodes (Fig. 3). Therefore, *mir-231* may act in the intestine to positively regulate GO toxicity in nematodes.

### Overexpression of *mir-231* in the intestine induced a susceptible property to GO toxicity in nematodes

**Figure 4:**
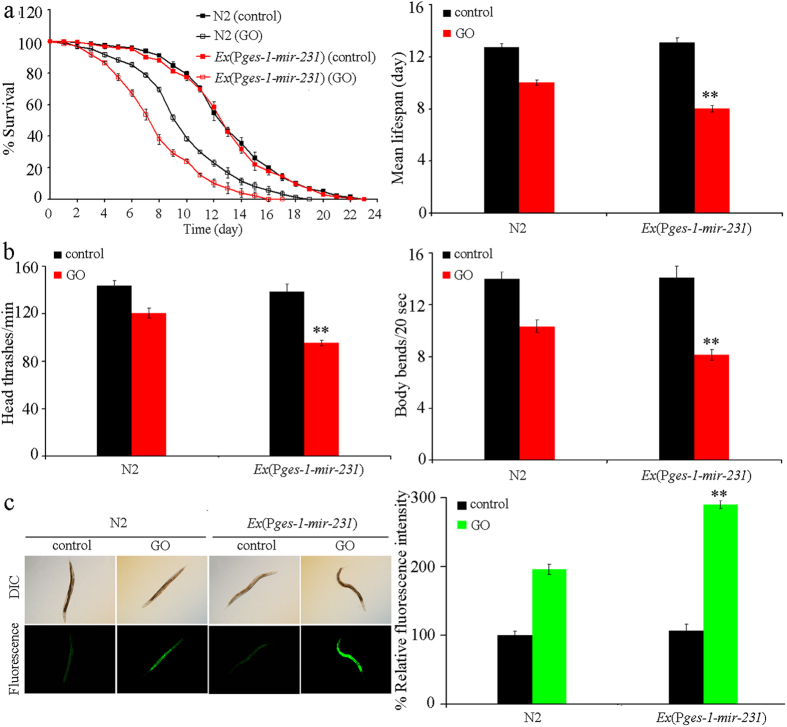
Effects of *mir-231* overexpression in intestine on GO toxicity in nematodes. (**a**) Effects of *mir-231* overexpression in intestine on GO toxicity in reducing lifespan in nematodes. (**b**) Effects of *mir-231* overexpression in intestine on GO toxicity in decreasing locomotion behavior in nematodes. (**c**) Effects of *mir-231* overexpression in intestine on GO toxicity in inducing intestinal ROS production in nematodes. GO exposure concentration was 100 mg/L. Prolonged exposure was performed from L1-larvae to young adults. Bars represent means ± SD. **P < 0.01 *vs* N2.

To confirm the intestine-specific activity of *mir-231* in positively regulating GO toxicity, we constructed the transgenic strain *Ex*(P*ges-1-mir-231*) which overexpresses *mir-231* specifically in the intestine. Overexpression of *mir-231* in this transgenic strain was confirmed by assessing the levels of *mir-231* transcription (Fig. S1). Transgenic strain of *Ex*(P*ges-1-mir-231*) had the similar phenotypes of lifespan, locomotion behavior, and intestinal ROS production to those in wild-type nematodes (Fig. 4). Moreover, GO (10 mg/L) exposed transgenic strain of *Ex*(P*ges-1-mir-231*) exhibited more severe reduction in lifespan, decrease in locomotion behavior, and induction of intestinal ROS production than GO (100 mg/L) exposed wild-type nematodes (Fig. 4). These results suggest that *mir-231* overexpression in the intestine can induce a susceptible property to GO toxicity in nematodes.

### *smk-1* might act as a potential targeted gene for *mir-231* in nematodes

**Figure 5:**
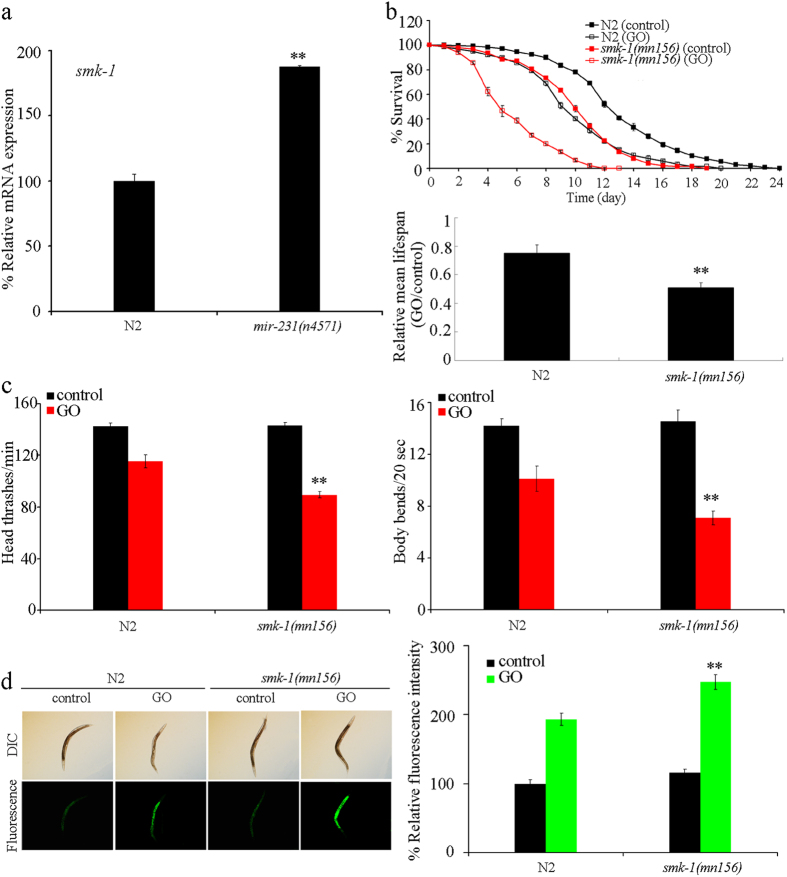
Effects of *smk-1* mutation on GO toxicity in nematodes. (**a**) Effect of *mir-231* mutation on expression of *smk-1* gene. (**b**) Effects of *smk-1* mutation on GO toxicity in reducing lifespan in nematodes. (**c**) Effects of *smk-1* mutation on GO toxicity in decreasing locomotion behavior in nematodes. (**d**) Effects of *smk-1* mutation on GO toxicity in inducing intestinal ROS production in nematodes. GO exposure concentration was 100 mg/L. Prolonged exposure was performed from L1-larvae to young adults. Bars represent means ± SD. **P < 0.01 *vs* N2.

We identified 93 putative *mir-231* targeted genes including *smk-1* using the TargetScan tool. The biological functions of the predicted targeted genes for *mir-231* were either unknown or associated with the development in nematodes. *mir-231* was predicted to act as an upstream regulator for *smk-1* by binding its 3′-UTR. The expression of *smk-1* gene was significantly higher in the loss-of-function *mir-231*(*n4571*) mutant than in wild-type nematodes (Fig. 5a), implying that *mir-231* may suppress the expression of *smk-1* gene in nematodes.

### SMK-1 confered protection against GO toxicity in nematodes

We next investigated the role of SMK-1 in GO susceptibility in nematodes using the *smk-1*(*mn156*) mutant. In the absence of GO, the *smk-1*(*mn156*) mutant had a reduced lifespan, normal locomotion behavior, and no significant induction of intestinal ROS production (Fig. 5b–d). After prolonged exposure to GO (100 mg/L), *smk-1*(*mn156*) mutant showed the more severe reduction in lifespan, decrease in locomotion behavior, and induction of intestinal ROS production than wild-type nematodes (Fig. 5b–d). Since the *smk-1*(*mn156*) mutant was more susceptible to GO toxicity than wild-type nematodes, SMK-1 appears to confer protection against GO toxicity in nematodes.

### Genetic interaction between *mir-231* and *smk-1* in regulating GO toxicity in nematodes

**Figure 6:**
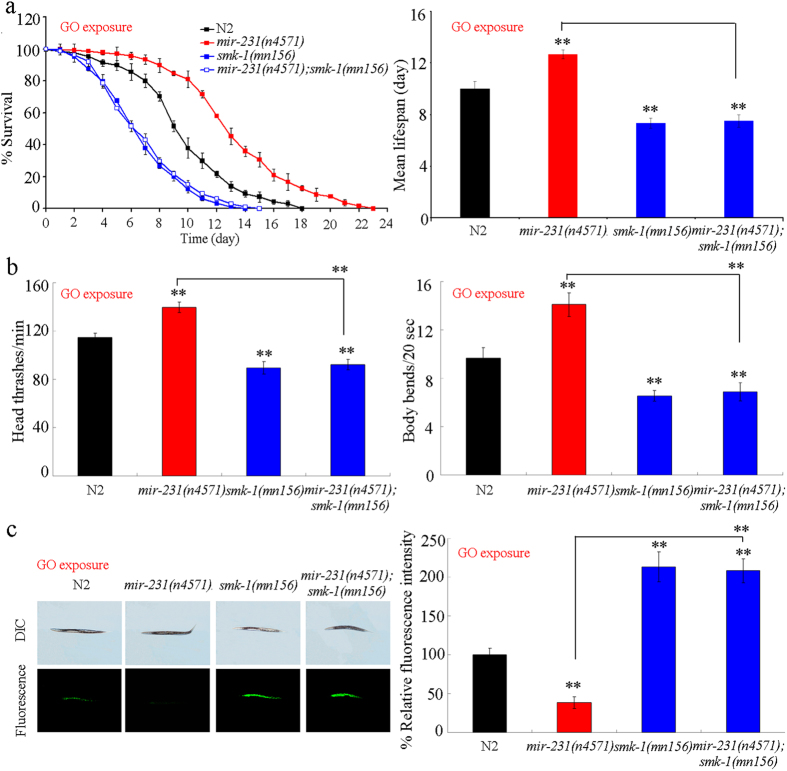
Genetic interaction between *mir-231* and *smk-1* in regulating GO toxicity in nematodes. (**a**) Genetic interaction between *mir-231* and *smk-1* in regulating GO toxicity in reducing lifespan in nematodes. (**b**) Genetic interaction between *mir-231* and *smk-1* in regulating GO toxicity in decreasing locomotion behavior in nematodes. (**c**) Genetic interaction between *mir-231* and *smk-1* in regulating GO toxicity inducing intestinal ROS production in nematodes. GO exposure concentration was 100 mg/L. Prolonged exposure was performed from L1-larvae to young adults. Bars represent means ± SD. **P < 0.01 *vs* N2 (if not specially indicated).

To assess the interaction between *mir-231* and *smk-1* in regulating GO toxicity, we compared the GO toxicity in double mutant of *mir-231*(*n4571*)*;smk-1*(*mn156*) with that in single mutant of *mir-231*(*n4571*) or *smk-1*(*mn156*). After exposure to GO (100 mg/L), the lifespan, locomotion behavior, and induction of intestinal ROS production in double mutant of *mir-231*(*n4571*)*;smk-1*(*mn156*) were similar to those in single mutant of *smk-1*(*mn156*) (Fig. 6), indicating that the GO resistance of the *mir-231*(*n4571*) mutant could be reversed by the loss of *smk-1* in nematodes. Therefore, *mir-231* may inhibit the ability of *smk-1* to protect against GO toxicity in nematodes.

### Tissue-specific activity of *smk-1* in regulating GO toxicity in nematodes

**Figure 7:**
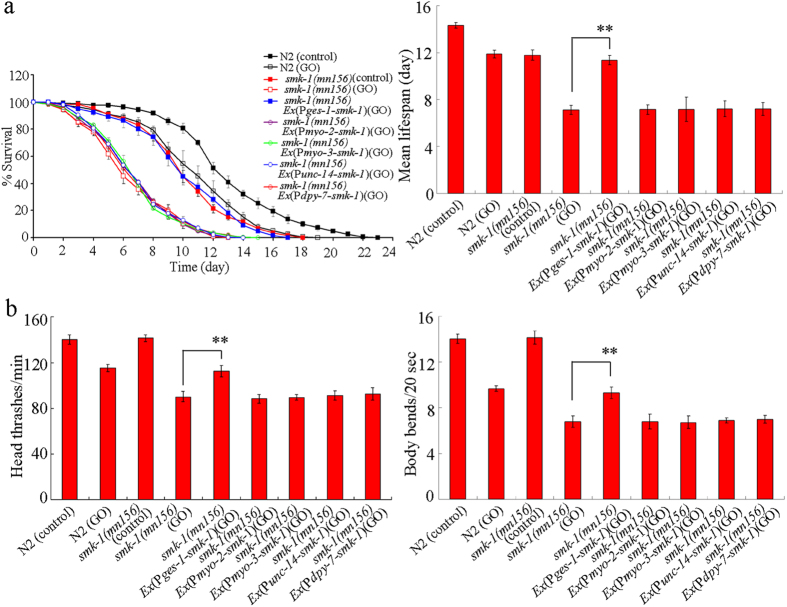
Tissue-specific activity of *smk-1* in regulating GO toxicity in nematodes. (**a**) Tissue-specific activity of *smk-1* in regulating GO toxicity on lifespan in nematodes. (**b**) Tissue-specific activity of *smk-1* in regulating GO toxicity on locomotion behavior in nematodes. GO exposure concentration was 100 mg/L. Prolonged exposure was performed from L1-larvae to young adults. Bars represent means ± SD. **P < 0.01.

In *C. elegans*, *smk-1* gene is expressed in intestine, pharynx, neurons, muscle, and hypodermis^23^. Using tissue-specific promoters, we investigated the tissue-specific activity of *smk-1* in negatively regulating GO toxicity in nematodes. The tissue-restricted expression of *smk-1* in the pharynx, neurons, muscle or hypodermis did not significantly influence the lifespan or locomotion behavior in *smk-1*(*mn156*) mutant nematodes exposed to GO (100 mg/L) (Fig. 7). However, expression of *smk-1* in the intestine significantly increased the lifespan and locomotion behavior in *smk-1*(*mn156*) mutant nematodes exposed to GO (100 mg/L) (Fig. 7). These results suggest that *smk-1* may also act in the intestine to protect against GO toxicity in nematodes.

### Intestinal overexpression of *smk-1* induced a resistant property to GO toxicity in nematodes

**Figure 8:**
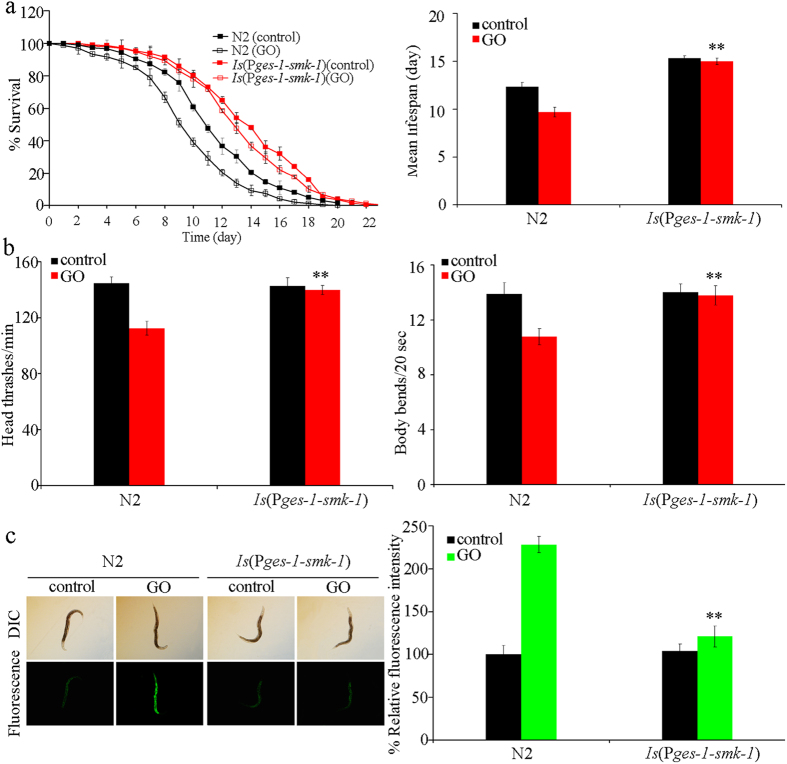
Effects of *smk-1* over expression in intestine on GO toxicity in nematodes. (**a**) Effects of *smk-1* overexpression in intestine on GO toxicity in reducing lifespan in nematodes. (**b**) Effects of *smk-1* overexpression in intestine on GO toxicity in decreasing locomotion behavior in nematodes. (**c**) Effects of *smk-1* overexpression in intestine on GO toxicity in inducing intestinal ROS production in nematodes. GO exposure concentration was 100 mg/L. Prolonged exposure was performed from L1-larvae to young adults. Bars represent means ± SD. **P < 0.01 *vs* N2.

To further characterize the intestine-specific activity of *smk-1* in negatively regulating GO toxicity, we constructed the transgenic strain *Is*(P*ges-1-smk-1*) which overexpresses *smk-1* specifically in the intestine. The transgenic strain of *Is*(P*ges-1-smk-1*) had an increased lifespan compared with wild-type nematodes, but had the similar locomotion behavior and intestinal ROS production to those in wild-type nematodes (Fig. 8). Exposure to GO (100 mg/L) did not affect the lifespan of the transgenic strain *Is*(P*ges-1-smk-1*) (Fig. 8a). Moreover, the locomotion behavior and intestinal ROS production in GO-treated *Is*(P*ges-1-smk-1*) nematodes were comparable to those in wild-type or untreated *Is*(P*ges-1-smk-1*) nematodes (Fig. 8b,c). Therefore, our results suggest that the intestinal overexpression of *smk-1* can induce resistance to GO toxicity in nematodes.

### Genetic interaction between *smk-1* and *daf-16* in regulating GO toxicity in nematodes

**Figure 9:**
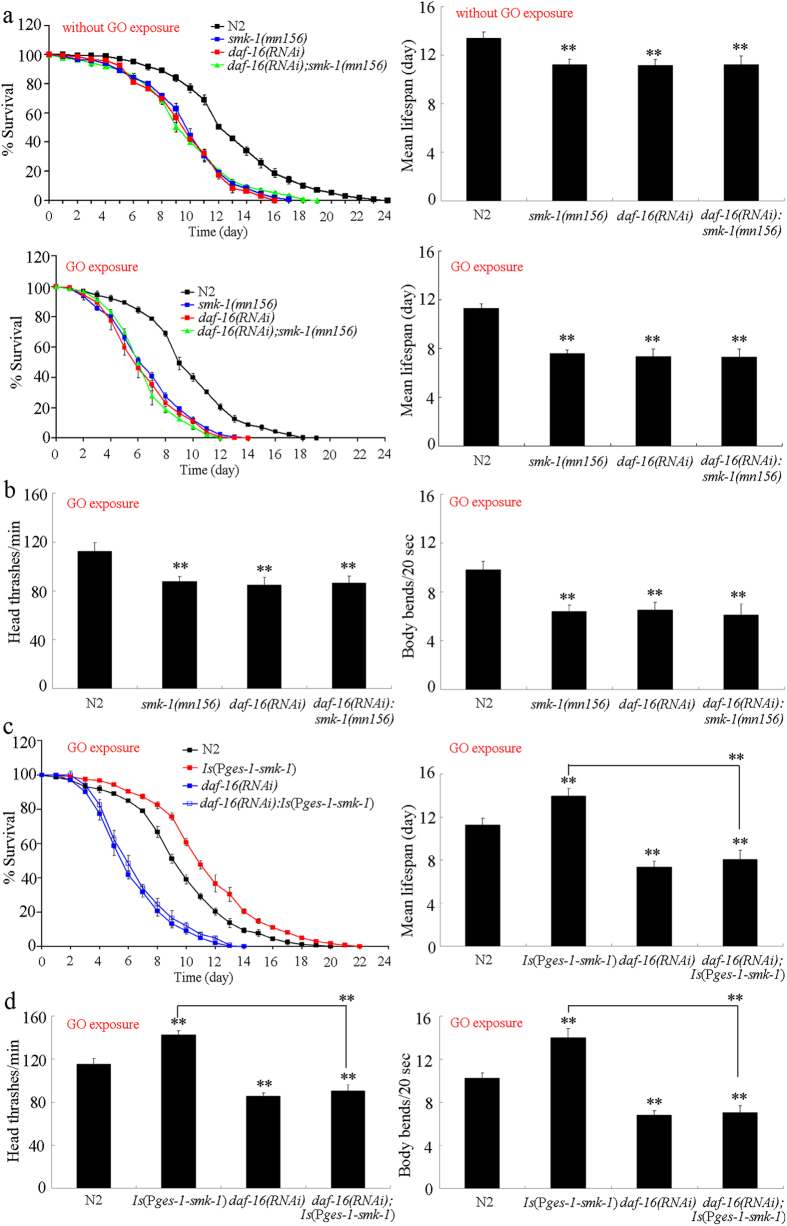
Genetic interaction between *smk-1* and *daf-16* in regulating GO toxicity in nematodes. (**a**) Genetic interaction between *smk-1* and *daf-16* in regulating GO toxicity in reducing lifespan in nematodes. (**b**) Genetic interaction between *smk-1* and *daf-16* in regulating GO toxicity in decreasing locomotion behavior in nematodes. (**c**) Effect of RNAi knockdown of *daf-16* gene on lifespan in GO exposed transgenic nematodes overexpressing *smk-1* in intestine. (**d**) Effect of RNAi knockdown of *daf-16* gene on locomotion behavior in GO exposed transgenic nematodes overexpressing *smk-1* in intestine. GO exposure concentration was 100 mg/L. Prolonged exposure was performed from L1-larvae to young adults. Bars represent means ± SD. **P < 0.01 *vs* N2 (if not specially indicated).

An earlier study suggested a possible genetic interaction between *smk-1* and *daf-16* in regulating biological processes such as longevity^23^. Under normal condition, the lifespan of the double mutant of *daf-16*(*RNAi*)*;smk-1*(*mn156*) was similar to that in single mutant of *daf-16*(*RNAi*) or *smk-1*(*mn156*) (Fig. 9a). To determine the interaction between *smk-1* and *daf-16* in regulating GO toxicity, we compared the GO toxicity in the double mutant of *daf-16*(*RNAi*)*;smk-1*(*mn156*) with that in single mutant of *daf-16*(*RNAi*) or *smk-1*(*mn156*). After exposure to GO (100 mg/L), the lifespan and locomotion behavior in the double mutant of *daf-16*(*RNAi*)*;smk-1*(*mn156*) were similar to those in single mutant of *smk-1*(*mn156*) or *daf-16*(*RNAi*) nematodes (Fig. 9a,b). Therefore, both *smk-1* and *daf-16* are required to protect against GO toxicity and may act in the same genetic pathway in nematodes.

### SMK-1 acted upstream of DAF-16 to regulate GO toxicity in nematodes

To determine the order in which *smk-1* and *daf-16* act in regulating GO toxicity, we examined the effects of RNA interference (RNAi) knockdown of *daf-16* gene on lifespan and locomotion behavior in GO exposed transgenic nematodes overexpressing *smk-1* in the intestine. Interestingly, we found that the RNAi knockdown of *daf-16* gene significantly suppressed the protective effects of *smk-*1 overexpression on both the lifespan and the locomotion behavior of GO-exposed nematodes (Fig. 9c,d). These results suggest that *smk-1* may act upstream of *daf-16* to protect against GO toxicity in nematodes.

In addition, loss-of-function mutation of *daf-16* gene did not affect *mir-231* expression under normal conditions or in response to 100 mg/L GO (Fig. S2). These data imply that *mir-231* may not act downstream of the transcriptional factor DAF-16 to regulate biological events in nematodes.

## Discussion

In nematodes, GO exposure caused the decrease in both the transcriptional expression of *mir-231*^18^ and the *mir-231::GFP* in the pharynx, intestine, neurons, and hypodermis (Fig. 2). It has been shown that loss-of-function mutation of *mir-231* induced a resistant property of nematodes to GO toxicity (Fig. 3)^18^. These results imply that *mir-231* might encode an important molecular signaling in nematodes to protect against potential GO toxicity. Previous study has also suggested that *mir-231* expression was increased during late developmental stages in adult nematodes^24^, implying its involvement in the anti-aging protection mechanism in nematodes.

In nematodes, *mir-231* is expressed in several tissues including the intestine, pharynx, hypodermis, and neurons^21^. GO exposure could decrease the expression of *mir-231::GFP* in all these tissues, especially in the intestine (Fig. 2). Tissue-specific activity assays indicated that *mir-231* acted in the intestine to regulate the GO toxicity on lifespan and locomotion behavior in nematodes (Fig. 3). Intestinal barrier has been shown to play crucial roles in protecting nematodes from toxic ENMs such as quantum dots (QDs) or GO in nematodes^13^,^25^. Our data imply that *mir-231* may be involved in the control of intestinal signaling pathways in GO exposed nematodes. The increased sensitivity to GO toxicity in nematodes overexpressing *mir-231* in the intestine further confirmed this possibility (Fig. 4). Nevertheless, the potential functions of *mir-231* in the pharynx, hypodermis, and neurons are still unclear in nematodes.

Evidence suggests that miRNAs with lengths of about 22 nt may suppress the functions of targeted gene by inhibiting the translation of mRNAs by imprecise antisense base-pairing^26^. In this study, we raised several lines of evidence to demonstrate that *smk-1* may be a targeted gene for *mir-231* that functions to protect nematodes against GO toxicity. First, we observed that the expression of *smk-1* was increased in loss-of-function mutation of *mir-231* (Fig. 5a). Furthermore, in contrast to the phenotypes in GO-exposed *mir-231* mutant nematodes, GO-exposed *smk-1* mutants presented with increased GO sensitivity (Fig. 5b–d). It has also been shown that the *smk-1*(*mn156*) mutation results in enhanced radiosensitivity from proton microbeam exposure^27^. In addition, mutation of *smk-1* gene reversed the GO-resistant property of *mir-231* mutants (Fig. 6), and overexpression of *smk-1* lacking its 3′-UTR prevented the increased GO sensitivity of nematodes overexpressing *mir-231* (Fig. S3). Surprisingly, under normal condition, we found that the long-lived phenotype of nematodes overexpressing *smk-1* was not observed in *mir-231* mutant nematodes. However, it is possible that *mir-231* can regulate longevity through other yet to be identified targeted genes with different functions from *smk-1* in nematodes.

More importantly, genetic interaction assay suggested that SMK-1 and DAF-16 functioned in the same genetic pathway to regulate the GO toxicity (Fig. 9a,b). We further determined that DAF-16 acted downstream of SMK-1 to protect against GO toxicity in nematodes (Fig. 9c,d). An earlier study has described the importance of the SMK-1-DAF-16 signaling cascade in the control of longevity^23^. In the present study, our results further suggest a novel function of the SMK-1-DAF-16 signaling cascade in the control of nanotoxicity in nematodes.

**Figure 10:**
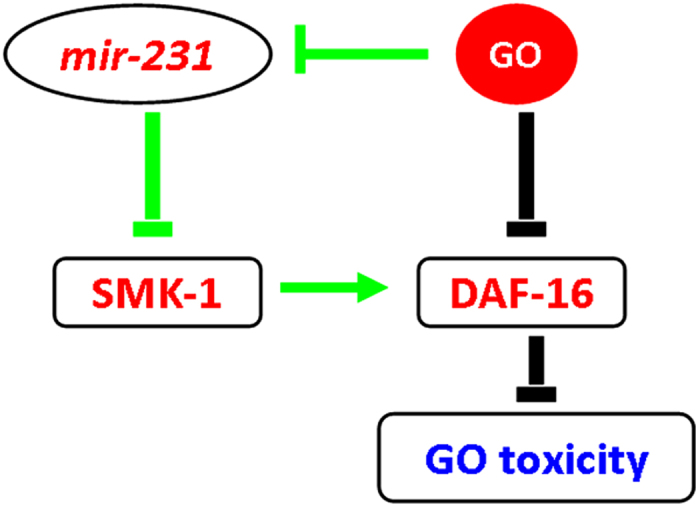
A diagram showing the *mir-231*-mediated molecular signaling in the control of GO toxicity in nematodes. Previous study has demonstrated the important function of DAF-16 in the control of GO toxicity in nematodes^29^.

Recently, it has been reported that GO exposure could result in the toxicity on nematodes by dysregulating functions of the intestinal insulin signaling pathway, and GO suppressed the expression of *daf-16* gene in nematodes^28^. Therefore, GO exposure may result in a novel dual regulation mechanism in the nematode intestine, a primary targeted organ for GO toxicity (Fig. 10). On the one hand, GO exposure can induce the toxic effects on lifespan, locomotion behavior, and intestinal function by suppressing the function of DAF-16 in the insulin signaling pathway. At the same time, GO exposure can activate a protection mechanism in nematodes by inhibiting the expression of *mir-231*. The inhibited expression of *mir-231* can lead to the activation of function of the SMK-1-DAF-16 signaling cascade, which in turn reduces GO toxicity in nematodes. This identified dual regulation mechanism implies that *mir-231* could be a potential candidate gene for the design of chemical modification or the selection of certain loaded drugs for GO for the aim of reducing the GO toxicity. Another dual regulation mechanism between *mir-360* and CEP-1 in the control of GO induced germline apoptosis was recently identified in the gonads, a secondary targeted organ of GO in nematodes^29^.

In conclusion, we investigated the *mir-231*-mediated molecular mechanisms underlying the response to GO exposure in *C. elegans*. We first identified the intestine-specific activity of *mir-231* in the regulation of GO toxicity. In the intestine, *mir-231* increased the effects of GO toxicity by suppressing the function of its target gene *smk-1*. SMK-1 acted upstream of DAF-16 in the insulin signaling pathway to protect against GO toxicity. Therefore, we discovered a new dual regulation mechanism between the *mir-231* and the SMK-1-DAF-16 signaling cascade in the control of GO toxicity in the intestine, the primary targeted organ of GO in nematodes. *C. elegans mir-231* an ortholog of human *miR-99* and *miR-556*^30^. Considering the extensive conservation of microRNAs in biology^30^, our results may lead to the discovery of important functions of *mir-231* and its homologues in regulating nanotoxicity in organisms.

## Methods

### Preparation of GO

GO was prepared from natural graphite powder according to the modified Hummer’s method^31^. After addition of graphite (2 g) and sodium nitrate (1 g) into a 250-mL flask, concentrated H_2_SO_4_ (50 mL) was added on ice, and KMnO_4_ (7 g) was further added. When temperature of the mixture warmed to 35 °C, H_2_O (90 mL) was slowly dripped into the paste. Diluted suspension was stirred at 70 °C for 15 min, and treated with a mixture of 7 mL of 30% H_2_O_2_ and 55 mL of H_2_O. The resulting warm suspension was filtered to obtain a yellow-brown filter cake, which was further washed with a solution of 3% HCl, followed by drying at 40 °C for 24 h. GO was obtained by ultrasonication of the as-made graphite oxide in water for 1 h.

### Characterization of GO

The prepared GO was characterized by atomic force microscopy (AFM, SPM-9600, Shimadzu, Japan), Raman spectroscopy using 632 nm wavelength excitation (Renishaw Invia Plus laser Raman spectrometer, Renishaw, UK), and zeta potential analyzed using a dynamic light scattering technique. To perform the AFM assay, GO suspension was pipetted on Si substrates, air-dried, and placed under AFM tip.

### *C. elegans* strains and exposure

Nematodes used were wild-type N2, mutants of *mir-231*(*n4571*), *smk-1*(*mn156*), *daf-16*(*mu86*), and *mir-231*(*n4571*)*;smk-1*(*mn156*), and transgenic strains of *maIs218*[*mir-231::GFP*], *Ex*(P*ges-1-mir-231*), *mir-231*(*n4571*)*Ex*(P*ges-1-mir-231*), *mir-231*(*n4571*)*Ex*(P*myo-2-mir-231*), *mir-231*(*n4571*)*Ex*(P*unc-14-mir-231*), *mir-231*(*n4571*)*Ex*(P*dpy-7-mir-231*), *Is*(P*ges-1-smk-1*), *smk-1*(*mn156*)*Ex*(P*ges-1-smk-1*), *smk-1*(*mn156*)*Ex*(P*myo-2-smk-1*), *smk-1*(*mn156*)*Ex*(P*myo-3-smk-1*), *smk-1*(*mn156*)*Ex*(P*unc-14-smk-1*), *smk-1*(*mn156*)*Ex*(P*dpy-7-smk-1*), and *Ex*(P*ges-1-mir-231*)*;Is*(P*ges-1-smk-1*). Some of them were from *Caenorhabditis* Genetics Center (funded by NIH Office of Research Infrastructure Programs (P40 OD010440)). Gravid nematodes were maintained on nematode growth medium (NGM) plates seeded with *Escherichia coli* OP50 at 20 °C^32^. Nematodes were lysed with a bleaching mixture (0.45 M NaOH, 2% HOCl) to obtain age synchronous L1-larvae populations as described^33^.

### Exposure and toxicity assessment

GO was sonicated for 30 min (40 kHz, 100 W), and then dispersed in K medium to prepare a stock solution (1 mg/mL). GO at the working concentration (100 mg/L) was prepared by diluting the stock solution with K medium. Prolonged exposure to GO was performed from L1-larvae to young adults in 12-well sterile tissue culture plates at 20 °C in the presence of food (OP50). After exposure, nematodes were used for the toxicity assessment using lifespan, locomotion behavior, and intestinal ROS production as the endpoints.

Lifespan was assayed at 20 °C basically as described^34^,^35^. During the lifespan assay, hermaphrodite nematodes were transferred daily for the first 7 days of adulthood. Nematodes would be checked every day, and were scored as dead if they did not move even after repeated taps with a pick. Sixty nematodes were examined per treatment, and three replicates were performed.

Endpoints of head thrash and body bend were used to reflect the locomotion behavior of nematodes as described^36^,^37^. Head thrash and body bend were assessed by under the dissecting microscope bye eyes. A head thrash is defined as a change in the direction of bending at the mid body. A body bend is defined as a change in the direction of the part of the nematodes corresponding to the posterior bulb of the pharynx along the *y* axis, assuming that nematode was traveling along the *x* axis. Twenty nematodes were examined per treatment, and six replicates were performed.

Intestinal ROS production was analyzed as described previously^38^,^39^. Intestinal ROS production reflects the functional state of intestine. The examined nematodes were transferred to 1 μM of 5′,6′-chloromethyl-2′,7′-dichlorodihydro-fluorescein diacetate (CM-H2DCFDA; Molecular Probes) to incubate for 3 h at 20 °C in the dark. Nematodes were then mounted on 2% agar pads for the examination at 488 nm of excitation wavelength and 510 nm of emission filter under a laser scanning confocal microscope (Leica, TCS SP2, Bensheim, Germany). Relative fluorescence intensity in intestine was semi-quantified, and the semiquantified ROS was expressed as relative fluorescence units (RFU) and normalized to autofluorescence. Twenty nematodes were examined per treatment, and six replicates were performed.

### Bioinformatics analysis for targeted gene prediction of *mir-231*

The corresponding targeted genes for *mir-231* were predicted using TargetScan version 6.2 (http://www.targetscan.org/worm_52/). TargetScan is a tool used for predicting biological targets of certain miRNA by searching for the presence of conserved sites that match seed region of a miRNA.

### Reverse-transcription and quantitative real-time polymerase chain reaction (qRT-PCR)

Total RNAs were extracted using RNeasy Mini kit (Qiagen), and reverse transcribed using PrimeScript ^TM^ RT reagent kit (Takara, Otsu, Shiga, Japan). After cDNA synthesis, real-time PCR was performed using SYBR Premix Ex Taq™ (Takara) for the amplification of PCR products. Real-time PCR was performed using primers for target gene of *smk-1* (forward primer, 5′-ATGTCGGACACAAAAGAGGT-3′; reverse primer, 5′-ATCCACCTGTTTTTCATCAA-3′), and reference gene of *tba-1* (forward primer, 5′-TCAACACTGCCATCGCCGCC-3′; reverse primer, 5′-TCCAAGCGAGACCAGGCTTCAG-3′). Real-time PCR was run at the optimized annealing temperature of 58 °C. Relative quantification of targeted gene in comparison to reference *tba-1* gene was determined, and the final results were expressed as relative expression ratio between targeted gene and reference gene. To analyze the transcriptional expression of *mir-231*, the primer used for the transcription of *mir-231* was GTCGTATCCAGTGCAGGGTCCGAGGTATTCGCACTGGATACGACTACAAG. The primer for qRT-PCR of *mir-231* was CTGACTGTTTCAAAAGCTTGTA, and the common reward primer was GTGCAGGGTCCGAGGT. All reactions were performed in triplicate.

### DNA constructs and germline transformation

To generate entry vector carrying promoter sequence, promoter region for *ges-1* gene specially expressed in intestine, *unc-14* gene specially expressed in neurons, *myo-3* gene specially expressed in muscle, *dpy-7* gene specially expressed in hypodermis, or *myo-2* gene specially expressed in pharynx was amplified by PCR from wild-type *C. elegans* genomic DNA. These promoter fragments were inserted into pPD95_77 vector in the sense orientation. *smk-1/F41E6.4a* cDNA or *mir-231* was amplified by PCR, and inserted into corresponding entry vector carrying the *ges-1*, *unc-14*, *myo-3*, *dpy-7*, or *myo-2* promoter sequence. Germline transformation was performed as described by coinjecting testing DNA at the concentration of 10–40 μg/mL and marker DNA of P*dop-1::rfp* at the concentration of 60 μg/mL into the gonad of nematodes^40^. Primer information for promoter amplification is shown in Table S1.

### RNAi

RNAi assay was performed by feeding animals with *E. coli* strain HT115 (DE3) expressing certain double-stranded RNA for *daf-16* gene as described^41^. *E. coli* HT115 (DE3) grown in LB broth containing ampicillin (100 μg/mL) was plated onto NGM containing ampicillin (100 μg/mL) and isopropyl 1-thio-β-D-galactopyranoside (IPTG, 5 mM). L2 larvae were transferred onto RNAi plates for 2 days until the nematodes became the gravid at 20 °C. Gravid adults were further transferred to fresh RNAi-expressing bacterial lawns to let them lay eggs for 2 h in order to obtain the second generation of RNAi population. Eggs were allowed to develop into young adults at 20 °C for the subsequent assays.

### Statistical analysis

Data in this article were expressed as means ± standard deviation (SD). Graphs were generated using Microsoft Excel software (Microsoft Corp., Redmond, WA). Statistical analysis was performed using SPSS 12.0 software (SPSS Inc., Chicago, USA). Differences between groups were determined using analysis of variance (ANOVA), and probability levels of 0.05 and 0.01 were considered statistically significant. The lifespan data were analyzed using a 2-tailed 2 sample *t*-test (Minitab Ltd, Coventry, UK).

## Additional Information

**How to cite this article**: Yang, R. *et al*. A *mir-231*-Regulated Protection Mechanism against the Toxicity of Graphene Oxide in Nematode *Caenorhabditis elegans.* *Sci. Rep.* **6**, 32214; doi: 10.1038/srep32214 (2016).

## Supplementary Material

Supplementary Information
