# Supplementary material for: A mir-231-Regulated Protection Mechanism against the Toxicity of Graphene Oxide in Nematode Caenorhabditis elegans
Source: Sci Rep. 2016 Aug 25;6:32214. doi: 10.1038/srep32214 (PMC4997325; doi:10.1038/srep32214)
Supplement: Supplementary Information [file srep32214-s1.doc]

**A *mir-231*-regulated Protection Mechanism against the Toxicity of Graphene Oxide in Nematode *Caenorhabditis elegans***

Ruilong Yang^1, 2^, Mingxia Ren^2^, Qi Rui^1, *^ & Dayong Wang^2, *^

^1^College of Life Sciences, Nanjing Agricultural University, Nanjing 210095, China.

^2^Key Laboratory of Environmental Medicine Engineering in Ministry of Education, Medical School, Southeast University, Nanjing 210009, China

^*^Correspondence and requests for materials should be addressed to Q.R. ([emai: lruiqi@njau.edu.cn](mailto:emai: lruiqi@njau.edu.cn)) and D.W. (e-mail: [dayongw@seu.edu.cn](mailto:dayongw@seu.edu.cn)).

**Supporting Information:**


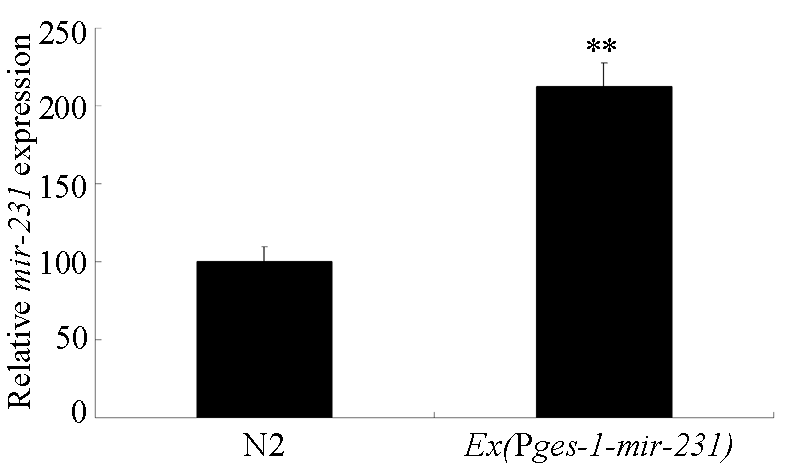


**Figure S1. Expression of *mir-231* in wild-type and transgenic strain of *Ex(*P*ges-1-mir-231)*.**  Bars represent means ± SD. ^**^*P* < 0.01 *vs* N2.


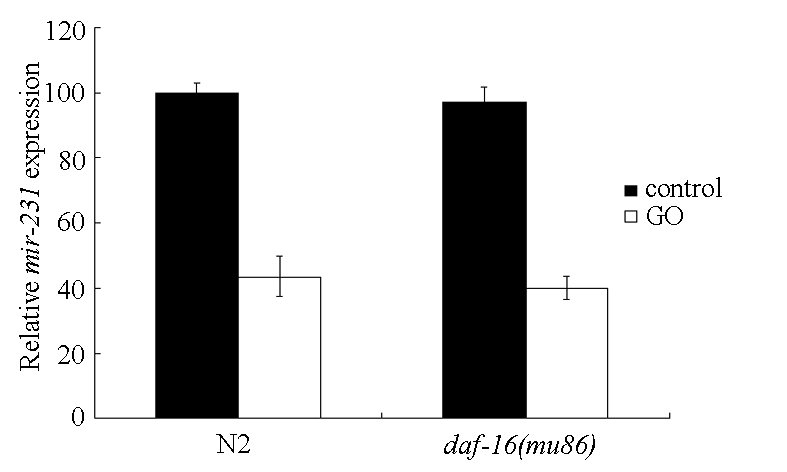


**Figure S2. Expression of *mir-231* in wild-type and *daf-16(mu86)* mutant nematodes.**  GO exposure concentration was 100 mg/L. Prolonged exposure was performed from L1-larvae to young adults. Bars represent means ± SD.


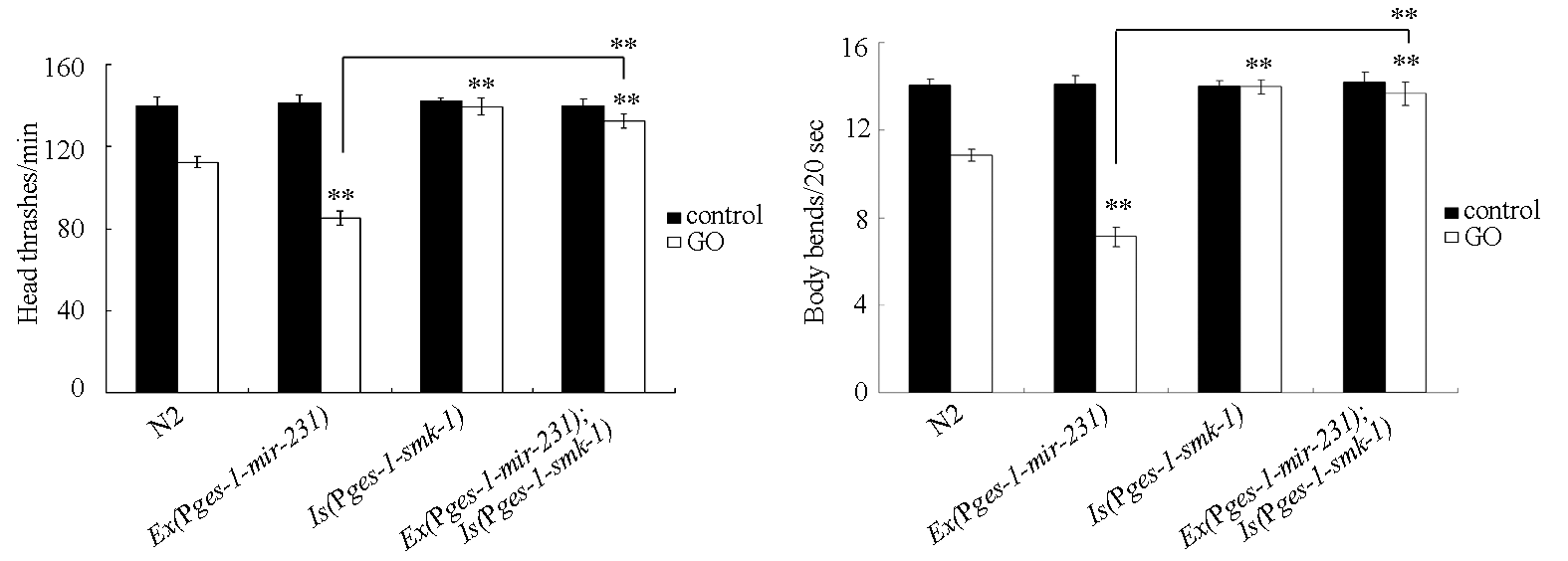


**Figure S3. Overexpression of *smk-1* lacking 3’-UTR suppressed the susceptible property of nematodes overexpressing *mir-231* to GO toxicity on locomotion behavior in nematodes.**  GO exposure concentration was 100 mg/L. Prolonged exposure was performed from L1-larvae to young adults. Bars represent means ± SD. ^**^*P* < 0.01 *vs* N2 (if not specially indicated).

**Table S1. Primer information for vector constructions**

| Gene | Forward primer (5’-3’) | Reverse primer(5’-3’) |
| --- | --- | --- |
| *Pges-1* | CGGTCTAGAGTTTGTTATCATTGTCCA | CATGGATCCCATCTGAATTCAAAGATA |
| *Pmyo-2* | CCCAAGCTTGGTGGTGGACAGTAACTGTCTGT | AGCTCTAGAATTTCTGTGTCTGACGATCGAGG |
| *Punc-14* | ACGAAGCTTTTCCCAACTGGCAATACT | ATACTGCAGCCACAAAAGTTGAGAGCA |
| *Pdpy-7* | TACAAGCTTCTATGTGCAATGTCACGTGGA | CGCGGATCCCTGGAACAAAATGTAAGAATA |
| *Pmyo-3* | CTCAAGCTTCACTTCCGGCGCCCTGAATCTAA | TAGGGATCCCATTTCTAGATGGATCTAGTGGTCGTGGG |
| *mir-231* | TGGGGATCCGTACCTATGAAAACAATTAT | TATCCCGGGGTAAAATATGAATGACACTT |
| *smk-1/F41E6.4a* | ATACCCGGGATGTCGGACACAAAAGAGGT | ATAGGTACCTTAGGCCTGCGAAACTGTGG |
